# Thermally Stable Bulk Heterojunction Prepared by Sequential Deposition of Nanostructured Polymer and Fullerene

**DOI:** 10.3390/polym9090456

**Published:** 2017-09-17

**Authors:** Heewon Hwang, Hoyeon Lee, Shafidah Shafian, Wooseop Lee, Jeesoo Seok, Ka Yeon Ryu, Du Yeol Ryu, Kyungkon Kim

**Affiliations:** 1 Department of Chemistry and Nano Science, Ewha Womans University, Seoul 03760, Korea; lydiahwang91@ewhain.net (H.H.); shafidahshafian@ewhain.net (S.S.); zsoo@ewhain.net (J.S.); ryuky@ewhain.net (K.Y.R.); 2 Department of Chemical and Bimolecular Engineering, Yonsei University, Seoul 03722, Korea; hoyeon@yonsei.ac.kr (H.L.); leewooseop@yonsei.ac.kr (W.L.); dyryu@yonsei.ac.kr (D.Y.R.)

**Keywords:** organic solar cell, sequential deposition, bulk heterojunction, stability

## Abstract

A morphologically-stable polymer/fullerene heterojunction has been prepared by minimizing the intermixing between polymer and fullerene via sequential deposition (SqD) of a polymer and a fullerene solution. A low crystalline conjugated polymer of PCPDTBT (poly[2,6-(4,4-bis-(2-ethylhexyl)-4H-cyclopenta [2,1-b;3,4-b′]dithiophene)-alt-4,7(2,1,3-benzothiadiazole)]) has been utilized for the polymer layer and PC_71_BM (phenyl-C_71_-butyric-acid-methyl ester) for the fullerene layer, respectively. Firstly, a nanostructured PCPDTBT bottom layer was developed by utilizing various additives to increase the surface area of the polymer film. The PC_71_BM solution was prepared by dissolving it in the 1,2-dichloroethane (DCE), exhibiting a lower vapor pressure and slower diffusion into the polymer layer. The deposition of the PC_71_BM solution on the nanostructured PCPDTBT layer forms an inter-digitated bulk heterojunction (ID-BHJ) with minimized intermixing. The organic photovoltaic (OPV) device utilizing the ID-BHJ photoactive layer exhibits a highly reproducible solar cell performance. In spite of restricted intermixing between the PC_71_BM and the PCPDTBT, the efficiency of ID-BHJ OPVs (3.36%) is comparable to that of OPVs (3.87%) prepared by the conventional method (deposition of a blended solution of polymer:fullerene). The thermal stability of the ID-BHJ is superior to the bulk heterojunction (BHJ) prepared by the conventional method. The ID-BHJ OPV maintains 70% of its initial efficiency after thermal stress application for twelve days at 80 °C, whereas the conventional BHJ OPV maintains only 40% of its initial efficiency.

## 1. Introduction

**Figure 1:**
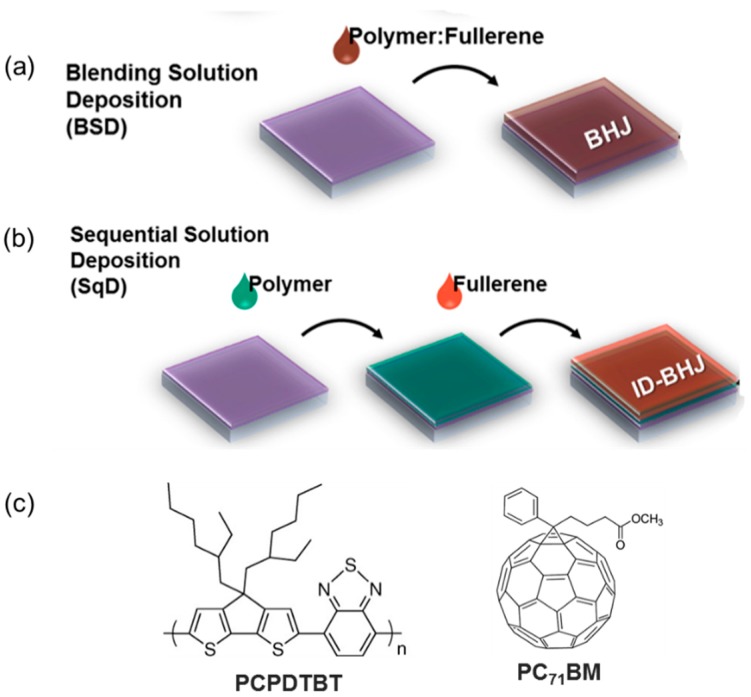
Schematic images of the bulk heterojunction formation methods: (**a**) blended solution deposition (BSD) (**b**) sequential solution deposition (SqD); and (**c**) chemical structures of poly[2,6-(4,4-bis-(2-ethylhexyl)-4H-cyclopenta [2,1-b;3,4-b′]dithiophene)-alt-4,7(2,1,3-benzothiadiazole)] (PCPDTBT) and phenyl-C_71_-butyric-acid-methyl ester (PC_71_BM). ID-BHJ, inter-digitated bulk heterojunction.

Recently, organic photovoltaics (OPVs) have exceeded 10% power conversion efficiency (PCE) by incorporating the finely-controlled bulk heterojunction (BHJ) photoactive layer morphology [1,2,3]. Due to the short exciton diffusion length of organic semiconductors, a fine and bi-continuous BHJ morphology is important for a high exciton dissociation efficiency and charge transport efficiency [4]. The most widely-utilized method to construct the BHJ morphology is the blended solution deposition (BSD) (Figure 1a), whereby the polymer and fullerene are dissolved in the same solvent and deposited to produce a BHJ layer. The optimum BHJ morphology can be developed by careful controlling the extent of the demixing between polymer and fullerene domains. Unfortunately, the optimized morphology is usually a kinetically-frozen state in which thermodynamic equilibrium has not been reached. This implies that the demixing would proceed further when the thermal stress is applied to the optimized BHJ morphology, resulting in poor thermal stability of the BHJ morphology, especially for the low crystalline polymers [5,6].

One way to overcome this limitation is forming the inter-digitated BHJ (ID-BHJ) morphology via the sequential deposition (SqD) of polymer and fullerene (Figure 1b) [7,8,9,10,11]. For constructing thermally-stable ID-BHJ by minimizing the intermixed phase of polymer and fullerene, a polymer layer should be deposited first on a substrate to have an ordered structure without disturbance of the bulky fullerene, and a fullerene layer should be deposited on the polymer layer by minimizing the intermixing. The small heterojunction area due to the minimized intermixing should be addressed.

Another point to be considered is securing the reproducibility of OPV prepared by the SqD process (SqD-OPV). Due to the limited heterojunction area of the photo-active layer fabricated by the SqD method, the performance of the SqD-OPV critically depends on the proper swelling of the fullerene into the polymer layer. The swelling process is sensitive to the fabrication conditions, such as the temperature and the kinds of solvents. Currently, highly volatile dichloromethane (DCM) is mainly utilized for the PCBM solvent in the SqD method, which makes SqD-OPV sensitive to the fabrication conditions.

In this work, we have addressed the above requirements by fabricating a nanostructured poly[2,6-(4,4-bis-(2-ethylhexyl)-4H-cyclopenta [2,1-b;3,4-b′]dithiophene)-alt-4,7(2,1,3-benzothiadiazole)] (PCPDTBT) (Figure 1c) bottom layer with a proper additive and utilizing 1,2-dichloroethane (DCE), exhibiting a lower vapor pressure and slower diffusion into the polymer layer, as the solvent for phenyl-C_71_-butyric-acid-methyl ester (PC_71_BM). We selected PCPDTBT because it has low crystallinity due to its bulk side chain, and OPV based on PCPDTBT exhibits poor stability. The deposition of PC_71_BM dissolved in the DCE onto the nanostructured PCPDTBT polymer bottom layer formed ID-BHJ with minimized intermixing. When the ID-BHJ was applied as a photoactive layer of the OPV, it exhibited superior morphological stability compared to the conventional BHJ.

## 2. Experimental Section

### 2.1. Device Fabrication

ID-BHJ devices were fabricated using the SqD method [8,9]. Pre-patterned 20 Ω/sq resistive indium tin oxide (ITO) glass substrates were ultrasonically washed in ethyl alcohol, acetone and isopropyl alcohol (10 min each). After drying in a convection oven (80 °C), all cells were exposed to UV-ozone for 20 min. PEDOT:PSS (poly(3,4-ethylenedioxythiophene) doped with poly(styrene sulfonate)) (AI4083) solution was vortexed with methanol at a 1:1 ratio. The solution was deposited onto the substrates by spin coating and dried in a vacuum oven at 110 °C. The final film thickness was approximately 30–35 nm. Poly[2,6-(4,4-bis-(2-ethylhexyl)-4H-cyclopenta [2,1-b;3,4-b′]dithiophene)-alt-4,7(2,1,3-benzothiadiazole)] (PCPDTBT) was used for the polymer bottom layer. The molecular weight of PCPDTBT was 50 kDa with a polydispersity of 2.5. PCPDTBT (8 mg/mL; 1-material) solution was stirred in chlorobenzene (CB) overnight on a 60 °C hotplate and filtered with a 0.2-μm polyvinylidene fluoride (PVDF) syringe filter (Whatman, Pittsburgh, PA, USA). The polymer solution was spin cast at 2200 rpm on top of PEDOT:PSS. The polymer solutions with ordering agents (OA) contained 5/10/20 vol % of 1-chloronaphthalene (CN) (Sigma Aldrich, Seoul, Korea) or 1,8-diiodooctane (DIO) (TCI, Tokyo, Japan). The films were dried for 15 min in a vacuum oven at 110 °C and dried overnight in a vacuum chamber to fully remove remnant OA. The thickness for all PCPDTBT films was 30–35 nm. PC_71_BM (EM Index, Korea) was dissolved in dichloromethane (DCM, 5 mg/mL, Daejung, Korea) or anhydrous 1,2-dichloroethane (DCE, 10 mg/mL, Sigma Aldrich, USA). One vol % of diphenyl ether (DPE) (Sigma Aldrich, USA) was added to improve the coating quality of the PC_71_BM layer. Both solutions were spin coated onto a PCPDTBT layer at a speed of 1500 rpm. After deposition, the films were dried in a vacuum oven at 110 °C for 15 min. For the conventional BHJ devices, PCPDTBT and PC_71_BM (1:3) were blended in CB:DIO (97:3 (*v*/*v*)) with a 32-mg/mL concentration and filtered using a 0.2-μm PVDF filter. The blended solution was spin coated at 2500 rpm and dried. For the electron transporting layer, 0.1 wt % di(2-pyridyl)ketone-stabilized TiO_2_ nanoparticles (TiO_2_ NP) were dispersed in *n*-butanol (Daejung, Seoul, Korea), spin cast at 1500 rpm and then dried on a 70 °C hotplate [12]. Finally, a 100-nm Al electrode was thermally evaporated. No further post treatment was performed on either type of device. The area of active layer was 0.12 cm^2^ for all of the devices.

### 2.2. Film Characterization

The topography and thickness of the films were scanned using atomic force microscopy (AFM) (AFM5100N, HITACHI, Tokyo, Japan) in tapping mode. UV-visible absorption spectra were measured using a UV-2450 spectrometer (SHIMADZU, Tokyo, Japan). The solvent swelling experiment was performed using an ellipsometry technique (SE MG-1000, Nano-View Co., Seoul, Korea). The polymer film was spin coated directly onto UV-ozone-treated clean Si wafers. A thicker polymer film (80 nm) was used to prevent film dewetting and to improve the measurement accuracy. After film preparation, 6 mL of the fullerene solvent were poured into the solvent chamber. DCM and DCE solvents were each tested. Then, the prepared polymer film was placed on the stage inside the solvent chamber to allow for solvent-uptake for 60 min at around 25 °C. Two ellipsometric angles (Ψ and δ) were obtained by in situ measurements, and the Lorentz oscillator model was adopted to fit the raw data. For grazing-incidence wide-angle X-ray scattering (GIWAXS, Pohang, Korea) characterization of the polymer films, well-dissolved PCPDTBT (10 mg/mL) was spin cast onto a PEDOT:PSS-coated silicon wafer and completely dried for more than 24 h in a vacuum oven at 110 °C. Afterwards, they were characterized by a monochromated X-ray beam (wavelength, λ = 1.12 nm) at PLS-II (Beamline 9A U-SAXS) at Pohang Accelerator Laboratory (PAL) in Korea. The two-dimensional X-ray scattering patterns were recorded with a 2D CCD detector (Rayonix SX165, Pohang, Korea), and the X-ray irradiation time was 5–10 s. The sample-to-detector distance was approximately 225 mm. The incidence angle of the X-ray beam was set to 0.12°.

Photoluminescence (PL) experiments were conducted using Fluorolog 3–11 (Horiba Jobinyvon) with a high pass filter of 800 nm. Films were excited at 700 nm and an incident beam angle of 55°. To solely investigate the exciton dissociation effect, the films were prepared on top of UV-ozone-treated bare glass without any charge transporting layers followed by encapsulation with a glass cap.

### 2.3. Device Characterization

The current density–voltage (*J*–*V*) was measured under 1-Sun illumination (McScience K201 LAB50) using a Keithley 2400 source meter. An incident photon-to-current efficiency (IPCE) measurement was performed at short-circuit conditions (McScience K3100 EQX). For the hole mobility measurement, an ITO/PEDOT:PSS/PCPDTBT (80 nm)/Au (60 nm) hole-only device was prepared, and the field independent space-charge-limited current (SCLC) model was employed to calculate the vertical hole mobility of the polymer film [13]. A dielectric constant of ε_r_ = 2.7 was used [14]. *J*–*V* curves were measured in the dark at room temperature.

## 3. Results and Discussion

Usually, for the formation of the ID-BHJ with a large heterojunction area, the PC_71_BM must be intermixed with the polymer under-layer by swelling the PC_71_BM solution into the polymer layer without dissolving it [8,15,16]. Organic solvents with a reasonable solubility for PC_71_BM have been selected, and their relevant properties are listed in Table 1. The solubility of PCPDTBT in various solvents was determined by spin coating a solvent onto the PCPDTBT film. *T*_100kPa_ represents the temperature at which the solvent vapor pressure is 100 kPa. Even though chlorobenzene, chloroform, 1,2-dichlorobenzene and toluene have a good PC_71_BM solubility, they are not adequate to be used as the PC_71_BM solvent, for those solvents have good solubility for PCPDTBT (Figure S1 in the Supplementary Information). In contrast, DCE, DCM and diiodomethane (DIM) exhibited high orthogonality to the bottom layer because no UV absorption change was observed after washing with these solvents. However, PC_71_BM dissolved in DIM could not be coated because of the high surface tension (50.8 mN/m at 20 °C) and high T_100kPa_ [17]. Therefore, the solvent swelling tests for PCPDTBT were performed for DCE and DCM.

**Figure 2:**
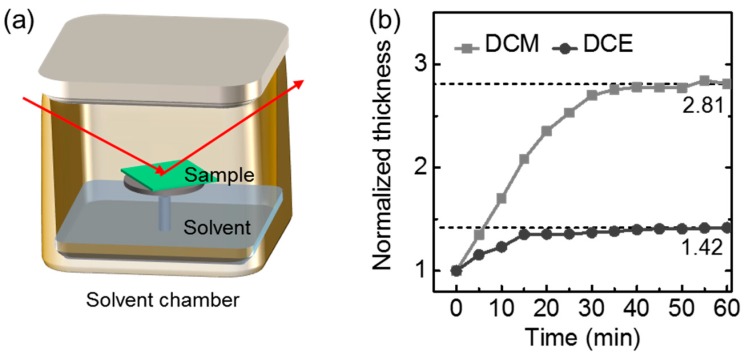
(**a**) Schematic view of the spectral ellipsometry experimental setup; and (**b**) the observed PCPDTBT saturation curve. Two different solvents (dichloromethane (DCM) and 1,2-dichloroethane (DCE)) were used. The initial film thickness was 80 nm.

PCPDTBT swelling by the corresponding solvent was monitored using a spectral ellipsometry apparatus (Figure 2a). Spectral ellipsometry is an adequate tool to monitor the solvent uptake phenomenon of the film. For the solvent swelling test, the thickness of the polymer film was monitored as a function of the swelling time. The swelling of PCPDTBT by the solvents was saturated within 30 min. The swollen thickness of the polymer films was determined from the plateau at 25 °C; PCPDTBT swelled more in DCM than in DCE (Figure 2b). It is expected that the PCPDTBT/PC_71_BM heterojunction area prepared by the DCE would be smaller than that by DCE because the PC_71_BM inter-diffuses into the amorphous swollen part of the polymer layer [8].

It should be noticed that diphenyl ether (DPE) was used as a processing additive to enhance the solubility and prevent the PC_71_BM aggregation. Those kinds of additives can reduce the fullerene aggregation size in solution and help form a conformal layer when deposited [9,16]. The atomic force microscopy (AFM) image of the PC_71_BM film topography on top of PCPDTBT (Figure S3 in the Supplementary Information) reveals that the addition of 1 vol % DPE can effectively reduce the PC_71_BM domain size and produce uniform films.

**Figure 3:**
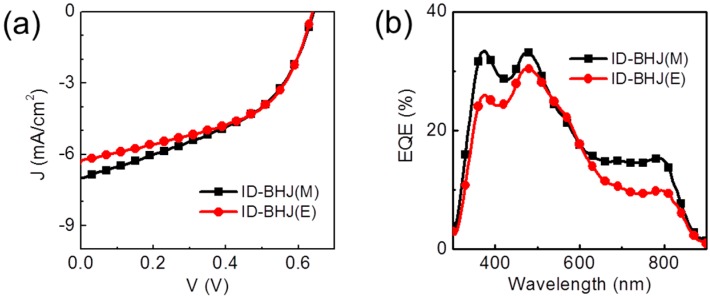
(**a**) The current density–voltage (*J*–*V*) curves for the devices; (**b**) external quantum efficiency (EQE) spectra of the ID-BHJ devices. M, DCM; E, DCE.

The performances of solar cells based on the ID-BHJ prepared by different PC_71_BM solvents were investigated. Table 2 and Figure 3 show the current density–voltage (*J*–*V*) characteristics and the incident photon-to-current efficiency (IPCE) spectra for the devices. For convenience, ID-BHJs prepared with PC_71_BM in DCE and DCM solutions will be denoted as ID-BHJ(E) and ID-BHJ(M), respectively. The photo current density (*J*_SC_) of ID-BHJ(M) was 10% larger than that of ID-BHJ(E). Based on the external quantum efficiency (EQE) spectrum, the *J*_SC_ difference was mainly ascribed to the different EQE values at the wavelength between 650 and 800 nm, which corresponds to the main absorption range of PCPDTBT. Since the DCM has a better polymer swelling ability, it can penetrate deeper into the PCPDTBT layer to create a larger polymer-fullerene heterojunction than DCE; thus, more excitons will be dissociated, and more charge will be collected.

To demonstrate the relation between the PC_71_BM penetration depth and photocurrent generation, we fabricated an inverted configuration device and compared the *J*_SC_ values with that of a conventional device [20].

**Figure 4:**
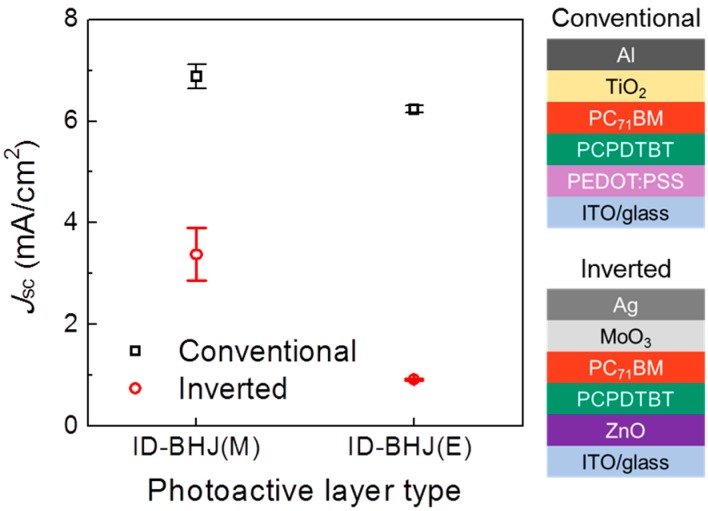
The current density (*J*_sc_) values are compared for different device configurations and PC_71_BM casting solvents.

To investigate the extent of the inter-diffusion of the PC_71_BM into the PCPDTBT bottom layer, the inverted-type device with the structure of ITO/ZnO/PCPDTBT/PC_71_BM/MoO_3_/Ag was fabricated, and its *J*_SC_ values were compared with the conventional-type device (Figure 4). The inverted-type device would not operate properly and would exhibit a low *J*_SC_ value if there were no intermixing between PCPDTBT and PC_71_BM; because holes (or electrons) in the PCPDTBT (or PC_71_BM) layer should travel through the PC_71_BM (or PCPDTBT) layer to be collected at the Ag (or ITO) electrode, which is not the proper direction for hole (or electron) transporting. However, when there is significant intermixing of PCPDTBT and PCBM, the inverted bilayer OPV would show a similar *J*_SC_ as the conventional-type device. The difference in *J*_SC_ between the conventional-type and inverted-type device (∆*J*_SC_) for the ID-BHJ(M) is much smaller than that of ID-BHJ(E). This implies that DCM assisted PC_71_BM in penetrating into the bottom polymer layer and formed BHJ throughout the film; however, the PC_71_BM dissolved in DCE could not penetrate far into the PCPDTBT layer.

DCM seems more suitable solvent than DCE for the formation of ID-BHJ. However, considering that the DCM is highly volatile, the formation of the ID-BHJ(M) would be influenced by the processing temperature significantly. Furthermore, obtaining a uniform PC_71_BM top layer would be difficult. The large standard deviation (SD) values of the ID-BHJ(M) (Table 2) indicate the low reproducibility and inhomogeneous film. We fabricated many ID-BHJ(M) films in different surrounding atmospheres and found that the film-forming process was highly sensitive to the temperature. A DCM-casted PC_71_BM did not form homogeneous layer on top of the PCPDTBT bottom layer at below room temperature, while DCE always produced a uniform PC_71_BM layer, regardless of the temperature (Figure S4 in the Supplementary Information). The SD value of the PCE of the ID-BHJ(E) was obtained as ±1.5% whereas that of ID-BHJ(M) was ±5.0%, which indicates that the performance of the ID-BHJ(E) is more reproducible. In addition, OPVs were fabricated at different temperatures to investigate the temperature dependence of solar cell performance. The solar performance of the ID-BHJ(E) was less dependent on the fabrication temperature compared to that of ID-BHJ(M) (Figure S4e in the Supplementary Information). The SD value of PCE of ID-BHJ(M) was significantly increased to ±15.8% when the processing temperature was increased to 60 °C. In contrast, the SD value of the PCE of ID-BHJ(E) was only ±1.1% when it was fabricated at the same temperature. The value was similar to the value of the ID-BHJ(E) fabricated at room temperature. This clearly showed that the solar cell performance of the DCE based OPV was less affected by the various fabrication conditions, which is an essential requirement for the commercialization of OPV.

Although DCE provided a high film quality and reproducibility, the small heterojunction area due to the poor swelling property of DCE should be addressed. We built a nano-morphology on the surface of PCPDTBT and deposited PC_71_BM by taking advantage of the low swelling property of DCE to minimize the intermixing and preserve the nanostructure of the PCPDTBT. There are several ways to induce the nano-morphology on the polymer layer, including nano-imprinting, lithography and self-assembly [21,22,23]. We used an ordering agent (OA) to evolve the nanostructured polymer surface, because a high boiling point OA reduces the solvent evaporation time and enhances the ordering of the polymer chains [9,24]. Two OAs with different polymer solubilities, polymer-soluble 1-chloronaphthalene (CN) and polymer-insoluble 1,8-diiodooctane (DIO), were used to study the nano-morphology evolution of PCPDTBT. Films that were prepared by PCPDTBT solution without OA, with CN and with DIO were denoted as PCPDTBT(N), PCPDTBT(C) and PCPDTBT(D), respectively. The three different volumes (5/10/20 vol % OA in chlorobenzene (CB)) were denoted as C5, C10, C20, D5, D10 and D20 for simplicity.

**Figure 5:**
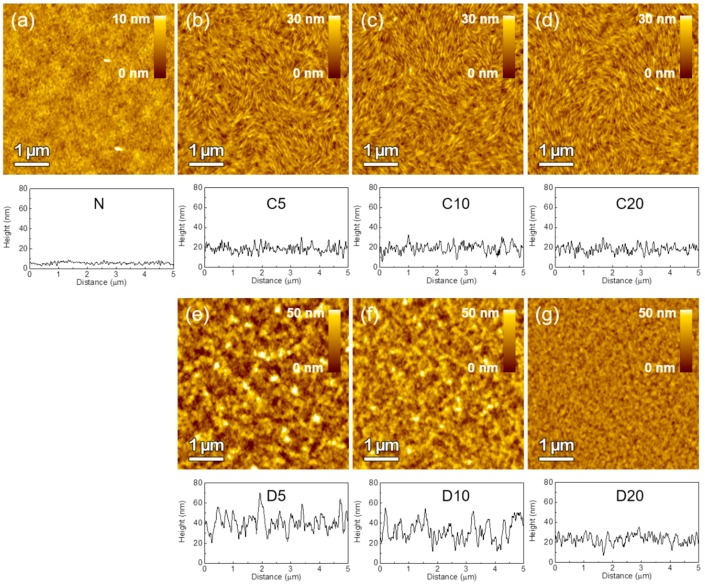
Atomic force microscopy images of the ordering agent (OA)-induced polymer surface morphology and height profile. PCPDTBT (**a**) without OA; (**b**) with 5 vol % 1-chloronaphthalene (CN); (**c**) with 10 vol % CN; (**d**) with 20 vol % CN; (**e**) with 5 vol % 1,8-diiodooctane (DIO); (**f**) with 10 vol % DIO; and (**g**) with 20 vol % DIO.

**Figure 6:**
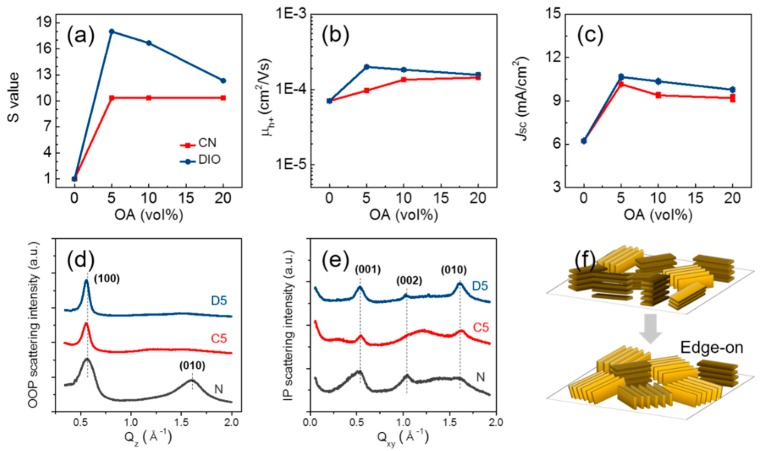
Various PCPDTBT films characterized by (**a**) normalized surface area increment (S value); (**b**) hole mobility (μ_h+_); and (**c**) *J*_SC_ versus OA volume; the grazing-incidence wide-angle X-ray scattering (GIWAXS) line-cuts in the (**d**) out-of-plane (OOP) direction and (**e**) in-plane (IP) direction; (**f**) proposed PCPDTBT film orientation change after OA addition.

Figure 5 shows the AFM-scanned polymer topography and height profile. The addition of OA roughened the polymer surface drastically by increasing the root-mean-square roughness value from 1.16 nm–4.33 nm for PCPDTBT (C5) and to 10.8 nm for PCPDTBT (D5), which resulted in increased surface area. We defined the S value to quantitatively compare the increased surface area; the S value is a normalized surface area increment obtained from the AFM measurement. Figure 6a displays the S value versus OA volume. OA-roughened films had 10–20-times larger S values than the PCPDTBT(N) film. OA not only roughened the polymer surface, but it also produced a unique nano-morphology. PCPDTBT(C) films have a nano-fibril-like morphology, while PCPDTBT(D) films have a rough morphology. Interestingly, a high-resolution image (Figure S5 in the Supplementary Information) of the CN-driven morphology reveals that a fibril is composed of several circular polymer domains in a row. The exact mechanism for domain formation is unknown, but Liu et al. proposed that some high boiling point additives can promote polymer aggregation in solution and form fibril-like aggregates while spin coating [25]. It is suggested that the different chemical structure of the OAs is the major cause of the different nano structures on the polymer surface. The CN has a benzene ring-based structure, which is similar to the chlorobenzene host solvent and polymer main chain, while DIO has no conjugated rings. Therefore, it is believed that the CN-containing solvent would have stronger π–π interactions with the polymer main chain than the DIO-containing solvent did, which provided the formation of the distinct fibril array morphology.

To investigate the crystalline structure of the nanostructured polymer films, a grazing-incidence wide-angle X-ray scattering (GIWAXS) study was performed using the PCPDTBT films on a PEDOT:PSS-coated silicon wafer. Figure 6d,e shows the out-of-plane (OOP) and in-plane (IP) line-cut profiles of PCPDTBT(N), PCPDTBT(C5) and PCPDTBT(D5) (the corresponding diffraction patterns and profiles of the other PCPDTBT films with different OA volumes can be found in Figures S6 and S7 in the Supplementary Information). Table 3 summarizes the diffraction peak analysis results for each film. The peak position values (*q*) and full-width at half-maximum values (∆*q*) are consistent with other studies [17,25]. In the OOP X-ray profiles, the PCPDTBT(N) film shows a peak at *q*_z_ ≈ 1.57 Å^−1^, corresponding to the π–π stacking reflection of (010). However, in both PCPDTBT(C) and PCPDTBT(D) films, the (010) intensity becomes much weaker, implying that no π–π stacking exists along the direction normal to the substrate. Instead, the ∆*q* value for the (100) peak (inter-chain separation within lamellae) is reduced in the OOP profiles. The reduction of ∆*q* is related to the increase of the polymer domain size and crystallinity. In addition, the coherence length (ξ) increased from 28.0 nm–51.0 nm for C5 and to 61.3 nm for D5, implying a larger size of the ordered domains. In the IP line-cut profile, the intensity of the (010) plane became stronger after OA addition, and the coherence length increased in the IP direction. Moreover, the peak positions slightly shifted to higher values (*q*_xy_ ≈ 1.62 Å^−1^), corresponding to a decrease in the π–π stacking spacing to ~3.89 Å for both PCPDTBT(C5) and PCPDTBT(D5). The (001) reflection in the IP profile refers to the spacing distances along the polymer backbone direction and shows similar changes as for (010). Overall, the GIWAXS profile reveals that both CN and DIO incorporation into the PCPDTBT solution significantly enhanced the polymer ordering in the edge-on direction after spin coating, while the PCPDTBT(N) film has mixed face-on and edge-on orientations (Figure 6f).

A hole mobility measurement was performed to investigate the influence of the film crystallinity on the hole mobility. The space-charge-limited current (SCLC) model is commonly used to calculate the hole mobility (μ_h+_) in the vertical direction in the PCPDTBT film. The calculated mobility (Table 4) is analogous to the other reference values [26,27]. Figure 6b is a plot of the hole mobility versus OA volume. Although the addition of OA increased the crystallinity of the polymer film in the GIWAXS result, it did not provide a large influence on the vertical hole mobility. Since the direction of π–π stacking was not normal to the substrate (face-on direction), the improved π–π stacking in polymer had not resulted in the enhancement of charge transport.

ID-BHJ structured films showing minimized intermixing were prepared by spin coating PC_71_BM(E) onto roughened polymer films. The absorbance of the polymer films was measured (Figure S8 in the Supplementary Information) to show that the addition of OA did not influence the film thickness nor absorption. AFM measurements revealed that the PCPDTBT film thickness was about 30–35 nm for all variables and that no morphological changes occurred after PC_71_BM(E) deposition due to the limited swelling of DCE into the polymer bottom layer (Figure S9 in the Supplementary Information). This implies that the surface morphology created on the polymer surface will be retained after forming heterojunctions with the PC_71_BM(E) layer.

To investigate whether the ID-BHJ could dissociate excitons effectively, we have investigated the PL quenching experiments for the ID-BHJ(D5) and ID-BHJ(C5) films (Figure S10 in the Supplementary Information). As expected, both films exhibited perfect PL quenching, which indicates that the morphology of the ID-BHJ structure was sufficient for the efficient exciton dissociation.

OPV devices were fabricated by using the ID-BHJ as a photoactive layer to demonstrate the influence of the bottom layer’s surface roughness on the device performances. Table 4 compares the solar cell performances of ID-BHJ devices with different OAs and BHJ devices. The ID-BHJ(D) and ID-BHJ© devices show better solar cell performances than ID-BHJ(N), which is mainly attributed to *J*_SC_ enhancement. As shown in Figure 6c, the change of the S value matches the change of the *J*_SC_ value, and ID-BHJ(D5) and ID-BHJ(C5) that have the highest S values exhibited the highest *J*_SC_ values of ~10.66 mA/cm^2^ and 10.16 mA/cm^2^. The PCE increased by about 72.3% for D5 addition compared to ID-BHJ(N). Although PCPDTBT(N) had the most favorable face-on orientation that is proper for facile charge transport, the ID-BHJ device incorporated with the edge-on orientation developed PCPDTBT(C5) and PCPDTBT(D5) showed larger values for *J*_SC_ by nearly a factor of two. Those results suggest that enhancing the surface area of the polymer bottom layer through the evolution of nanostructured morphology is the major factor influencing solar cell performances rather than polymer chain orientation. In other words, the heterojunction area is more important than the ordering of the polymer chain in fabricating the PCPDTBT/PC_71_BM ID-BHJ by using the low swelling DCE as the solvent for PC_71_BM.

**Figure 7:**
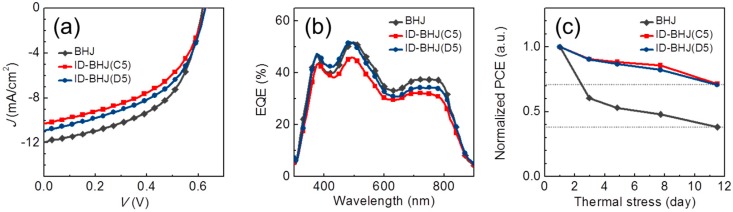
The device performances of BHJ, ID-BHJ(C5) and ID-BHJ(D5) compared in (**a**) a current density–voltage (*J–V*) curve; (**b**) external quantum efficiency (EQE) spectra; and (**c**) long-term thermal stability (compared on a hotplate at 80 °C for more than 10 days).

The best ID-BHJ devices are compared to the BHJ device in Figure 7 and Table 4. The optimization procedure for the BSJ device is included in the Supplementary Information (Table S1) to explain the inferior device performance compared to the reported results [28,29,30,31]. Both ID-BHJ devices showed comparable performances to that of BSJ in the *J–V* and EQE measurements. In particular, in the EQE spectra, the ID-BHJ(D) and ID-BHJ(E) had almost the same spectral shape, and the EQE value ofID-BHJ(D) and ID-BHJ(E) at 650–800 nm was greater than that of the ID-BHJ(N) (Figure 3). These results reflect that the heterojunction area of the ID-BHJ is similar with that of BHJ prepared by the conventional BSD method in spite of minimized intermixing.

From a morphological point of view, the morphology of the ID-BHJ photoactive layer, formed by the SqD process, was expected to be stable during the thermal stress test because the intermixing between the PCPDTBT and the PC_71_BM was minimized by the prevention of PC_71_BM diffusion into the pre-formed ordered PCPDTBT bottom layer; whereas, the PC_71_BM could be remained in the polymer domains in the conventional BHJ, and the demixing would proceed further when the thermal stress was applied to the optimized BHJ morphology, resulting in the poor thermal stability of the BHJ morphology, especially for the low crystalline polymers.

The thermal stability of the ID-BHJ device (Figure 7c) was tested on an 80 °C hotplate for more than 12 days. The stability result indicated that the ID-BHJ architecture was significantly superior to that of the conventional BHJ under the thermal stress condition. The conventional BHJ device retained only ~40% of its initial efficiency after 12 days, while both types of ID-BHJ devices retained ~70% of their initial efficiencies. To prove more clearly that the morphology of the ID-BHJ layer is stable under the thermal annealing, the topologies of the PCPDTBT bottom layer after selective removal of the PCBM layer from the ID-BHJ before and after thermal annealing were compared. As shown in the Figure S11 in the Supplementary Information, the surface morphology of the PCPDTBT layer remained almost unchanged even after 12 days of thermal annealing at 80 °C. This result clearly supports the superior morphological stability of the ID-BHJ.

## 4. Conclusions

A nanostructured PCPDTBT layer and PC_71_BM dissolved in the less swelling solvent of DCE were applied to construct the ID-BHJ morphology with minimized intermixing of the polymer and PC_71_BM. The performance of the ID-BHJ-based OPVs was more reproducible than for those that used PC_71_BM dissolved in highly volatile DCM. The ID-BHJ OPVs exhibited a superior morphological stability during thermal stress than conventional BHJ-based OPVs. We believe that the nanostructured polymer bottom layer and less volatile DCE could make the SqD-processed OPVs applicable to large-scale coating methods, such as slot die and bar coating.

## Figures and Tables

**Table 1:** Possible PC_71_BM solvents for the fabrication of the polymer/fullerene heterojunction.

Solvent	PC_71_BM Solubility (mg/mL) [18]	PCPDTBT Solubility	*T*_100kPa_^a^ (°C) [19]
Chlorobenzene	60.6	Good	131.3
Chloroform	61.1	Good	60.8
1,2-Dichlorobenzene	203.0	Good	180.0
1,2-Dichloroethane ^b^ (anhydrous)	15.4	Poor	83.1
Dichloromethane	4.47^9^	Poor	39.3
Diiodomethane	356.8	Poor	181.6
Toluene	27.4	Good	110.1

^a^ T_100kPa_ is the temperature at which the solvent vapor pressure is 100 kPa. ^b^ See the Supplementary Information (Figure S2) for the procedure to determine the PC_71_BM solubility.

**Table 2:** Photovoltaic parameters of organic photovoltaics (OPVs) with ID-BHJs prepared using different solvents for PC_71_BM.

Type	*V*_oc_ (V)	*J*_sc_ (mA/cm^2^)	FF (%)	PCE (%)
**ID-BHJ(M)**	0.63 ± 0.01	6.88 ± 0.24	43.7 ± 2.20	1.90 ± 0.10
**ID-BHJ(E)**	0.64 ± 0.00	6.25 ± 0.02	50.2 ± 0.91	2.00 ± 0.03

**Table 3:** Grazing-incidence wide-angle X-ray scattering (GIWAXS) diffraction peak analysis of PCPDTBT(N), PCPDTBT(C5) and PCPDTBT(D5) films.

OA	Index	*q* ^a^ (Å^−1^)	*d* ^b^ (Å)	∆*q* ^c^ (Å^−1^)	ξ ^d^ (Å)
**N**	OOP (100)	0.555	11.3	0.202	28.1
	OOP (010)	1.58	3.98	0.294	19.2
	IP (001)	0.489	12.9	0.199	28.4
	IP (010)	1.44	4.36	0.455	12.4
**C5**	OOP (100)	0.548	11.5	0.111	51.0
	IP (001)	0.538	11.7	0.0856	66.1
	IP (010)	1.62	3.89	0.184	30.8
**D5**	OOP (100)	0.549	11.4	0.0922	61.3
	IP (001)	0.532	11.8	0.0945	59.9
	IP (010)	1.62	3.89	0.140	40.3

^a^ Peak position; ^b^ spacing distance; ^c^ full-width at half-maximum; ^d^ coherence length, calculated using the Scherrer equation (ξ = 2π/∆*q*).

**Table 4:** Photovoltaic parameters and hole mobility of ID-BHJ OPVs using different polymer bottom layers.

Photoactive Layer Structure	PCPDTBT Bottom Layer	*V*_oc_ (V)	*J*_sc_ (mA/cm^2^)	Fill Factor (FF) (%)	PCE (%)	μ_h+_ (cm^2^/Vs)
**BHJ**	-	0.62 ± 0.00	11.88 ± 0.13	52.5 ± 0.19	3.87 ± 0.06	4.9 × 10^−5^
**ID-BHJ**	N	0.64 ± 0.00	6.23 ± 0.07	49.3 ± 0.86	1.95 ± 0.05	7.0 × 10^−5^
	C5	0.63 ± 0.00	10.16 ± 0.10	48.0 ± 0.00	3.06 ± 0.03	9.7 × 10^−5^
	C10	0.62 ± 0.00	9.39 ± 0.16	46.6 ± 0.73	2.72 ± 0.06	6.8 × 10^−5^
	C20	0.62 ± 0.00	9.19 ± 0.22	46.3 ± 0.48	2.62 ± 0.06	1.4 × 10^−4^
	D5	0.63 ± 0.00	10.66 ± 0.15	49.9 ± 0.25	3.36 ± 0.03	2.0 × 10^−4^
	D10	0.63 ± 0.00	10.35 ± 0.12	48.7 ± 0.09	3.15 ± 0.02	2.5 × 10^−4^
	D20	0.62 ± 0.00	9.77 ± 0.11	47.5 ± 0.11	2.90 ± 0.03	1.6 × 10^−4^

## Supplementary Materials

The following are available online at www.mdpi.com/2073-4360/9/9/456/s1: Figure S1: UV-absorption spectra of as-cast PCPDTBT film and after spin casting with each solvent. (**a**) Chlorobenzene (CB), (**b**) chloroform (CF), (**c**) o-dichlorobenzene (DCB), (**d**) 1,2-dichloroethane (DCE), (**e**) dichloromethane (DCM), (**f**) diiodomethane (DIM) and (**g**) toluene. Figure S2: (a) UV absorption spectra of the PC_71_BM solution with different concentrations. (b) The absorbance values obtained from 460 nm plotted with the concentration. The red star marks the absorbance of the diluted solution. Figure S3: Surface topography of (**a**) DCE-, (**b**) 1 vol % DPE-containing DCM- and (**c**) 1 vol % DPE-containing DCE-casted PC_71_BM films. Figure S4: DCM-casted PC_71_BM at (**a**) below RT and (**b**) 60 °C. DCE-casted PC_71_BM at (**c**) below RT (**d**) and 60 °C (**e**). PCE and *J*_SC_ are dependent on the processing temperature. Figure S5: The surface topography of ordering agent-driven PCPDTBT film scanned by an atomic force microscope (AFM). (1 × 1 μm). (**a**) PCPDTBT(N). (**b**) PCPDTBT(C5). (**c**) PCPDTBT(D5). Figure S6: Original grazing-incidence wide-angle X-ray scattering (GIWAXS) diffraction patterns of Figure 7d–e. (**a**) PCPDTBT(N). (**b**) PCPDTBT(C5). (**c**) PCPDTBT (D5). Figure S7: In-plane (IP) and out-of-plane (OOP) line-cuts from GIWAXS images. (**a**) IP and (**c**) OOP X-ray scattering profiles of PCPDTBT(C). (**b**) IP and (**d**) OOP X-ray scattering profiles of PCPDTBT(D). Figure S8: Absorption spectra of ordering agent (OA)-added PCPDTBT films. Figure S9: AFM-measured PCPDTBT film surface topography after PC_71_BM(E) removal from ID-BL. (**a**) PCPDTBT(N). (**b**) PCPDTBT(C5). (**c**) PCPDTBT(D5). Figure S10: Photoluminescence (PL) quenching effect of two different types of solar cells. Table S1: Optimization of device performance according to different active layer thicknesses.

## Abbreviations

AFM	atomic force microscopy
BHJ	bulk heterojunction
BSD	blended solution deposition
CB	chlorobenzene
CN	1-chloronaphthalene
DCE	1,2-dichloroethane
DCM	dichloromethane
DIM	diiodomethane
DIO	1,8-diiodooctane
DPE	diphenyl ether
EQE	external quantum efficiency
GIWAXS	grazing-incidence wide-angle X-ray scattering
ID-BHJ	inter-diffused bilayer
IP	in-plane
IPCE	incident photon-to-current efficiency
ITO	indium tin oxide
OA	ordering agent
OOP	out-of-plane
OPV	organic photovoltaics
PC_71_BM	phenyl-C_71_-butyric-acid-methyl ester
PCE	power conversion efficiency
PCPDTBT	poly[2,6-(4,4-bis-(2-ethylhexyl)-4H-cyclopenta [2,1-b;3,4-b′]dithiophene)-alt-4,7(2,1,3-benzothiadiazole)]
PEDOT:PSS	poly(3,4-ethylenedioxythiophene) doped with poly(styrene sulfonate)
PVDF	polyvinylidene fluoride
SCLC	space-charge-limited current
SqD	sequential deposition
