# Supplementary material for: Thermally Stable Bulk Heterojunction Prepared by Sequential Deposition of Nanostructured Polymer and Fullerene
Source: Polymers (Basel). 2017 Sep 17;9(9):456. doi: 10.3390/polym9090456 (PMC6418660; doi:10.3390/polym9090456)
Supplement: Supplementary file 1 [file polymers-09-00456-s001.pdf]

Supplementary Materials: Thermally Stable Bulk Heterojunction Prepared by Sequential Deposition of Nanostructured Polymer and Fullerene

Heewon Hwang, Hoyeon Lee, Shafidah Shafian, Wooseop Lee, Jeesoo Seok, Ka Yeon Ryu, Du Yeol Ryu and Kyungkon Kim

Contents

1. Optimization of blend single-layer device varying the layer thickness 1

2. Solvent orthogonality to polymer observation using UV spectrometer 2

3. Determination of $PC_{71}BM$ solubility in anhydrous 1,2-dichloroethane 3

4. Atomic force microscope scanned $PC_{71}BM$ film topography (5 × 5 μm) 3

5. Effect of surrounding atmosphere on $PC_{71}BM$ film formation 3

6. Atomic force microscope-scanned PCPDTBT film topography (1 × 1 μm) 4

7. Grazing-incidence wide-angle X-ray scattering analysis of PCPDTBT films 4

8. Absorption observation of ordering agent-added PCPDTBT films 5

9. Atomic force microscope-scanned PCPDTBT film topography (5 × 5 μm) after removing $PC_{71}BM$ from the ID-BHJ 5

10. Photoluminescence quenching effect 6

Reference 6

1. Optimization of Blend Single-Layer Device Varying the Layer Thickness

The blend single-layer (BSL) was optimized to compare the device performance of the inter-diffused bilayer (ID-BL) device. The PCPDTBT:$PC_{71}BM$ BSL solar cell was broadly studied by many researchers. Bazan's group reported the highest PCPDTBT:$PC_{71}BM$ solar cell efficiency of up to 5.5% by incorporating alkanedithiol processing additive[1]. Since PCPDTBT is known for its large batch-to-batch variation due to the complicated synthesis process[2], it is worth demonstrating the optimization process. We fabricated the BSL device based on the highest record device. The blend solution of PCPDTBT (1-material; Lot#SX7118) and $PC_{71}BM$ (EM Index) was prepared in 1:3 ratios in chlorobenzene (CB). For the processing additive, we used 3 vol% of 1,8-diiodooctane (DIO) instead of alkanedithiol. The final device configuration is as follows: ITO/PEDOT:PSS/PCPDTBT:$PC_{71}BM$/TiO_2/Al. Table S1 displays the average device performance of BSL. The 90 nm-thick BSL shows the highest power conversion efficiency (PCE) of 3.87%. Therefore, the BHJ device mentioned in this manuscript is fabricated using 90 nm-thick BSL.

Table S1. Optimization of device performance according to different active layer thicknesses.

Thickness (nm)	Concentration (mg/mL)	Spin coating (rpm)	V_{oc} (V)	J_{sc} (mA/cm²)	FF	PCE (%)
70	32	3500	0.61	11.68	0.51	3.63
80	32	3000	0.62	11.65	0.52	3.77
90	32	2500	0.62	11.88	0.53	3.87
100	40	3500	0.62	11.66	0.52	3.74

2. Solvent Orthogonality to Polymer Observation Using UV Spectrometer

Solvent orthogonality to the polymer layer was observed using UV-2450 (SHIMADZU) by comparing the absorption difference between as-cast PCPDTBT film and solvent-washed PCPDTBT film. In Figure S1, chlorobenzene (CB), chloroform (CF), o-dichlorobenzene (DCB) and toluene-washed polymer film show significantly reduced absorption due to high solubility on PCPDTBT; while 1,2-dichloroethane (DCE), dichloromethane (DCM) and diiodomethane (DIM) show high orthogonality to PCPDTBT film.

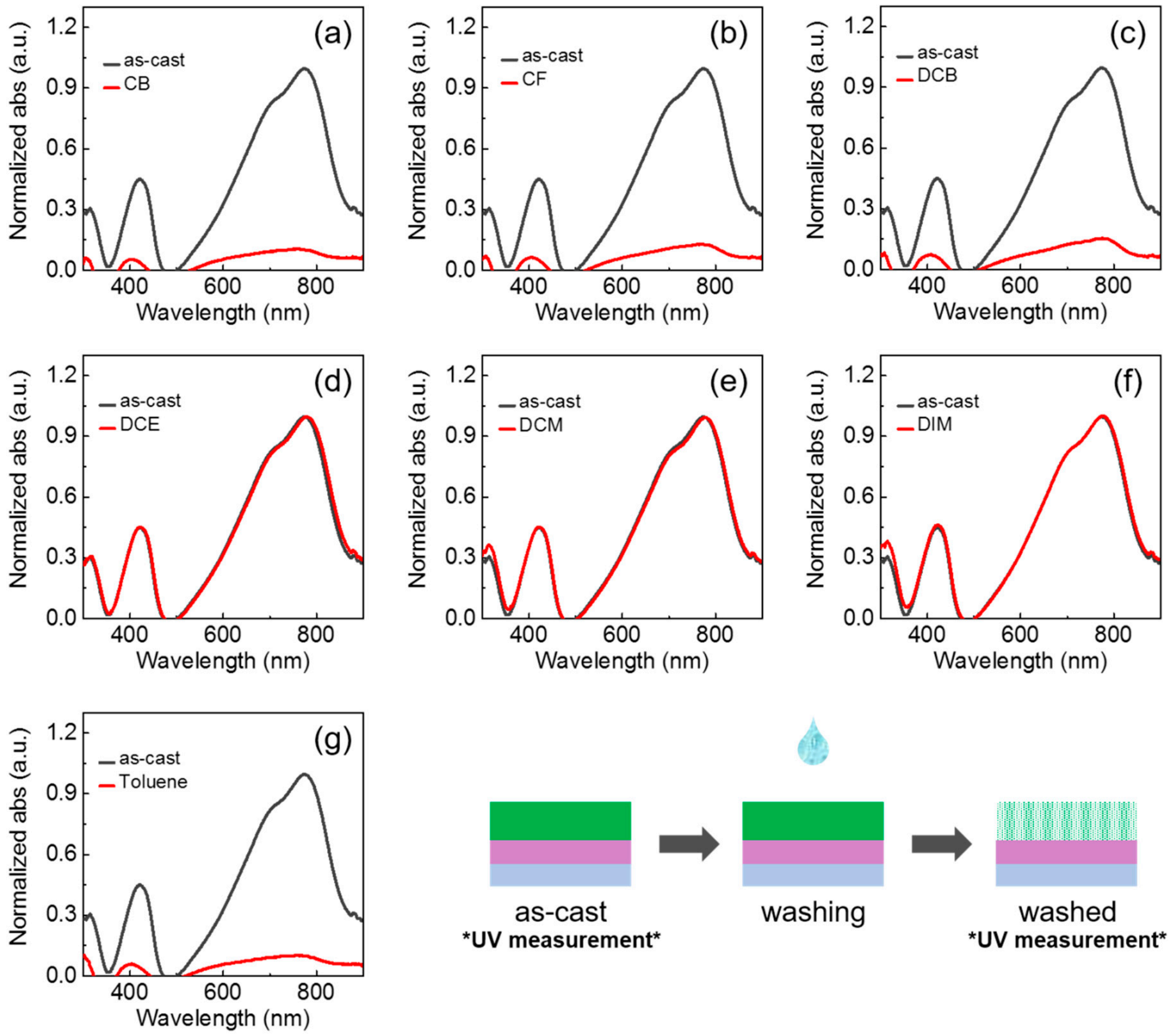

Figure S1. UV-absorption spectra of as-cast PCPDTBT film and after spin casting with each solvent. (a) Chlorobenzene (CB), (b) chloroform (CF), (c) o-dichlorobenzene (DCB), (d) 1,2-dichloroethane (DCE), (e) dichloromethane (DCM), (f) diiodomethane (DIM) and (g) toluene.

3. Determination of $PC_{71}BM$ Solubility in Anhydrous 1,2-Dichloroethane

The solubility of $PC_{71}BM$ in anhydrous DCE was determined using a UV-Vis spectrometer. The solute $PC_{71}BM$ was stirred in anhydrous DCE with 0.018 mg/mL,
0.036 mg/mL and 0.09 mg/mL concentrations for two hours before measurement. The unknown solution is the saturated solution diluted three hundred times. All of the solutions were filtered with a 1.0 μL nylon syringe filter (PALL) prior to measuring. The absorbance values are obtained from 460 nm and plotted in Figure S2. The unknown concentration is marked with a red star. Based on the linear plot, the unknown concentration is 15.4 mg/mL.

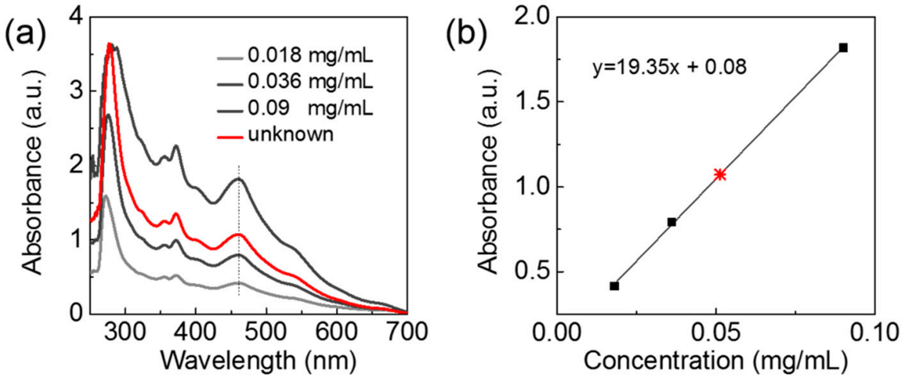

Figure S2. (a) UV absorption spectra of the $PC_{71}BM$ solution with different concentrations. (b) The absorbance values obtained from 460 nm plotted with the concentration. The red star marks the absorbance of the diluted solution.

4. Atomic Force Microscope-Scanned $PC_{71}BM$ Film Topography (5 × 5 μm)

The surface topography of various solvent-casted $PC_{71}BM$ films was scanned using an atomic force microscope (AFM) (AFM5100N, HITACHI). The addition of processing additive can enhance the solubility of $PC_{71}BM$ in organic solvents and prevent the formation of large aggregates inside the film. Zsoo et al. demonstrated the effect of processing additive in DCM previously[5]. High boiling point solvent diphenyl ether (T_{bp} = 258 °C) is used as the processing additive in this manuscript. In Figure S3, large $PC_{71}BM$ aggregates (~200 nm) are observed in DCE-only casted film, while 1 vol% DPE in the host solvent can significantly reduce the $PC_{71}BM$ domain size and produce fine quality films.

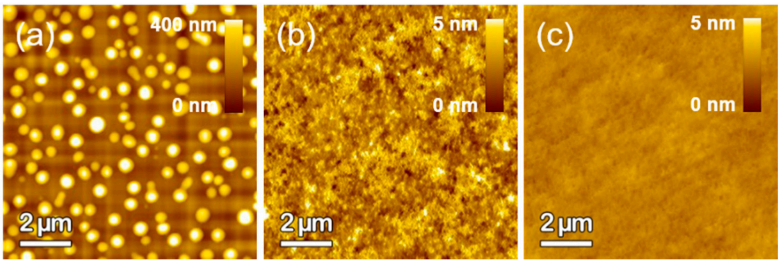

Figure S3. Surface topography of (a) DCE-, (b) 1 vol% DPE-containing DCM- and (c) 1 vol% DPE-containing DCE-casted $PC_{71}BM$ films.

5. Effect of the Surrounding Atmosphere on $PC_{71}BM$ Film Formation

Figure S4 shows images of $PC_{71}BM$ films spin coated with different base solvents at lower than RT and 60 °C. For temperature controlled, PCPDTBT-coated films were heated at the desired temperature on the hotplate prior to spin coating the $PC_{71}BM$ solution.

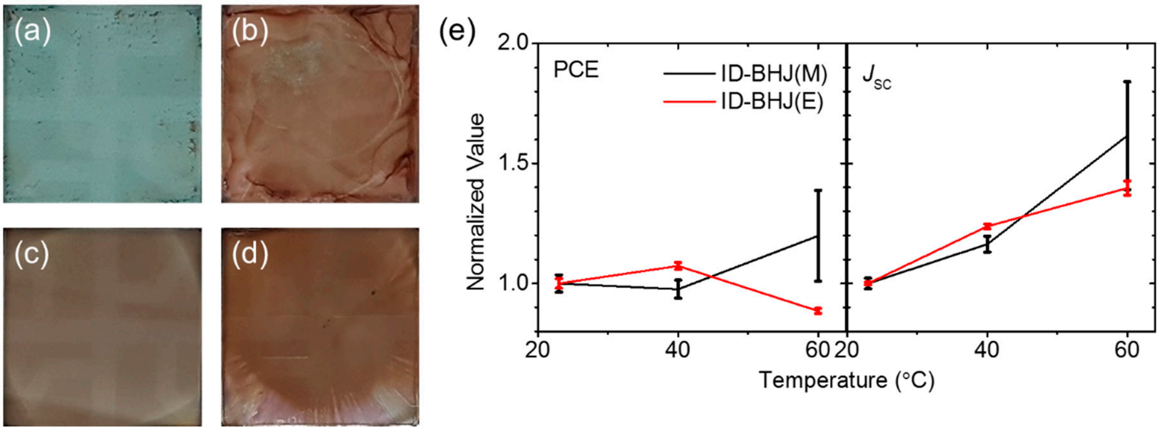

Figure S4. DCM-casted $PC_{71}BM$ at (a) below RT and (b) 60 °C. DCE-casted $PC_{71}BM$ at (c) below RT (d) and 60 °C (e). PCE and J_{SC} are dependent on the processing temperature.

6. Atomic Force Microscope-Scanned PCPDTBT Film Topography (1 × 1 µm)

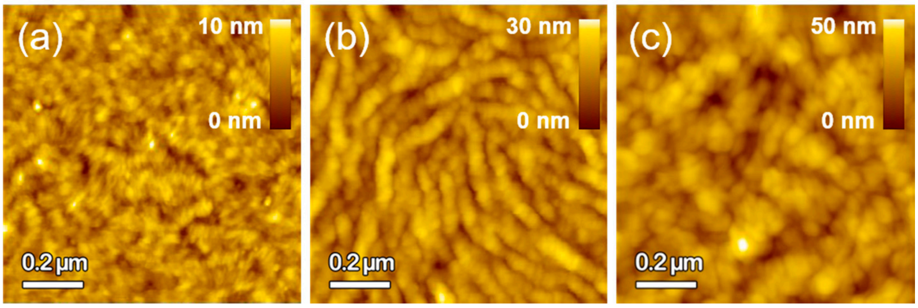

Figure S5. The surface topography of ordering agent-driven PCPDTBT film scanned by an atomic force microscope (AFM). (1 × 1 µm). (a) PCPDTBT(N). (b) PCPDTBT(C5). (c) PCPDTBT(D5).

7. Grazing-Incidence Wide-Angle X-Ray Scattering Analysis on PCPDTBT Films

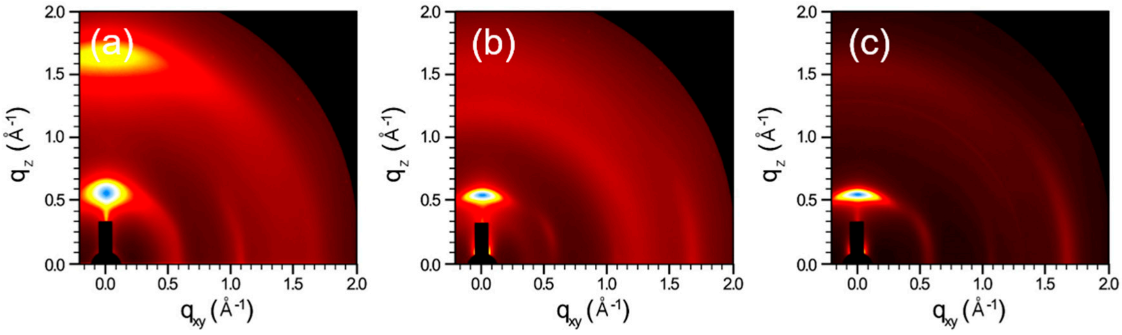

Figure S6. Original grazing-incidence wide-angle X-ray scattering (GIWAXS) diffraction patterns of Figure 7d–e. (a) PCPDTBT(N). (b) PCPDTBT(C5). (c) PCPDTBT (D5).

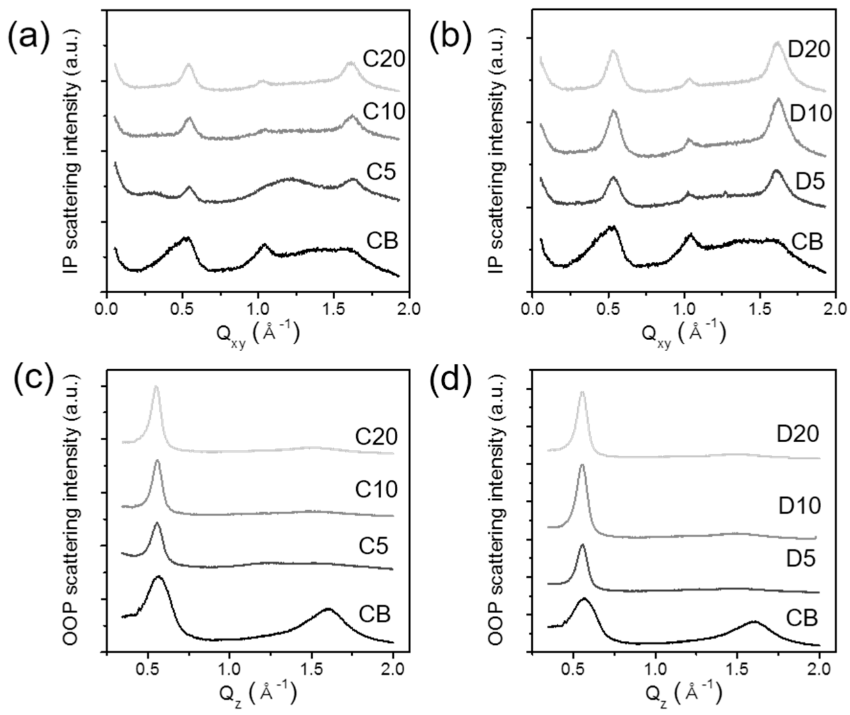

Figure S7. In-plane (IP) and out-of-plane (OOP) line-cuts from GIWAXS images. (a) IP and (c) OOP X-ray scattering profiles of PCPDTBT(C). (b) IP and (d) OOP X-ray scattering profiles of PCPDTBT(D).

8. Absorption Observation of Ordering Agent-Added PCPDTBT Films

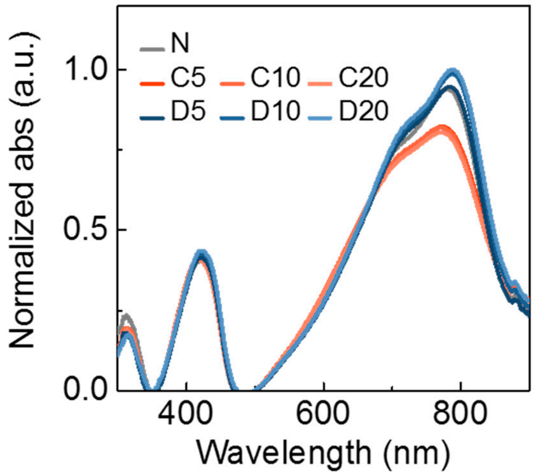

Figure S8. Absorption spectra of ordering agent (OA)-added PCPDTBT films.

9. Atomic Force Microscope-Scanned PCPDTBT Film Topography (5 × 5 μm) after Removing $PC_{71}BM$ from the ID-BHJ

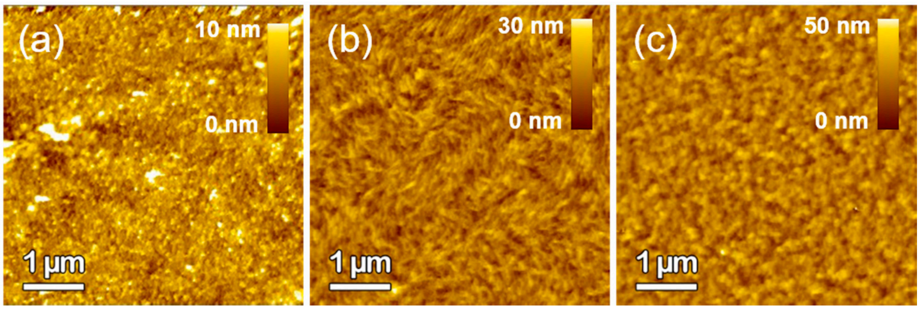

Figure S9. AFM-measured PCPDTBT film surface topography after $PC_{71}BM$(E) removal from ID-BL. (a) PCPDTBT(N). (b) PCPDTBT(C5). (c) PCPDTBT(D5).

10. Photoluminescence Quenching Effect

Photoluminescence quenching effects of two different types of solar cells were studied for additional morphological evidences. To solely observe the exciton dissociation effect by adding the n-type semiconductor on p-type polymer, the films were prepared on top of UV-ozone-treated bare glass without any charge transporting layers. The resulting graphs are presented in Figure S10. Regardless of the device structure and morphology, the fullerene addition results in perfect quenching, indicating that the exciton in the SqD-processed
ID-BHJ can be dissociated as efficiently as the conventional BHJ morphology.

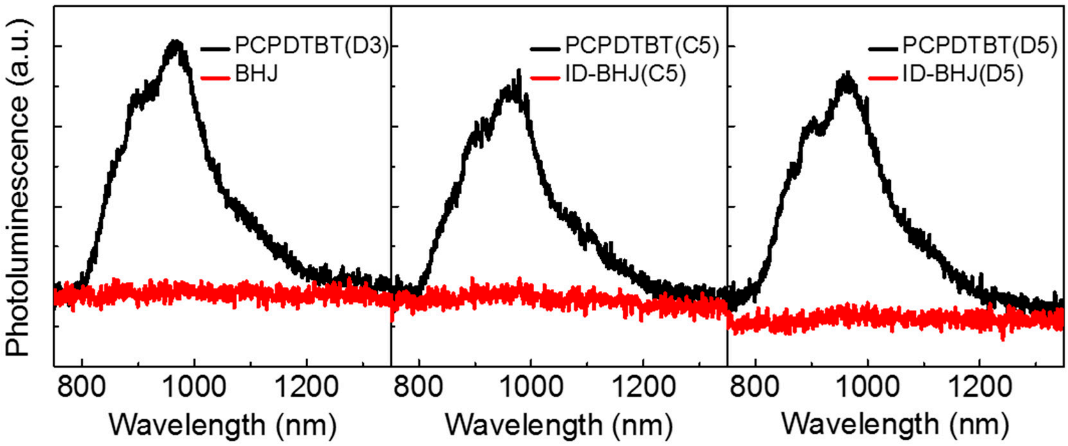

Figure S10. Photoluminescence (PL) quenching effect of two different types of solar cells.

Reference

1. Peet, J.; Kim, J. Y.; Coates, N. E.; Ma, W. L.; Moses, D.; Heeger, A. J.; Bazan, G. C., Efficiency enhancement in low-bandgap polymer solar cells by processing with alkane dithiols. *Nat Mater* 2007, *6* (7), 497-500.
2. Lee, J. K.; Ma, W. L.; Brabec, C. J.; Yuen, J.; Moon, J. S.; Kim, J. Y.; Lee, K.; Bazan, G. C.; Heeger, A. J., Processing additives for improved efficiency from bulk heterojunction solar cells. *Journal of the American Chemical Society* 2008, *130* (11), 3619-3623.
3. Machui, F.; Abbott, S.; Waller, D.; Koppe, M.; Brabec, C. J., Determination of Solubility Parameters for Organic Semiconductor Formulations. *Macromolecular Chemistry and Physics* 2011, *212* (19), 2159-2165.
4. Hansen, C. M., *Hansen solubility parameters: a user's handbook*. CRC press: 2007.
5. Seok, J.; Shin, T. J.; Park, S.; Cho, C.; Lee, J. Y.; Yeol Ryu, D.; Kim, M. H.; Kim, K., Efficient organic photovoltaics utilizing nanoscale heterojunctions in sequentially deposited polymer/fullerene bilayer. *Sci Rep* 2015, *5*, 8373.
